# The influence of the internet on choices about older adults’ health and well-being

**DOI:** 10.1590/0034-7167-2023-0321

**Published:** 2024-05-13

**Authors:** Cristina Braga, Karen Ruggeri Saad, Marcia Kiyomi Koike

**Affiliations:** I Instituto de Assistência Médica ao Servidor Público Estadual de São Paulo. São Paulo, São Paulo, Brazil; II Universidade Federal do Vale do São Francisco. Petrolina, Pernambuco, Brazil; III Universidade de São Paulo. São Paulo, São Paulo, Brazil

**Keywords:** Aged, Information Seeking Behavior, Health of the Eldery, Decsion Making, Forecasting, Idoso, Comportamento de Busca de Informação, Saúde do Idoso, Tomada de Decisões, Previsões, Anciano, Conducta en la Búsquda de Información, Salud del Anciano, Tomada de Decisiones, Predicción

## Abstract

**Objectives:**

to describe the profile of older adults who access the internet to search for health information and identify the factors that can influence older adults’ decisions about their health based on information collected online.

**Methods:**

391 older adults answered an online questionnaire regarding habits and satisfaction with information about health collected on the internet. Data processing involved Logistic Regression.

**Results:**

higher education reduces by 44% the likelihood of an older adult following the health recommendations on internet sites. However, social activities and self-perceived health increase the possibility of following the recommendations by 83% and 71%, respectively. The belief that the internet promotes healthy habits increases by 29.2 times the probability of an older adult following the advice.

**Final Considerations:**

knowing the profile of older adults who use the Internet can help professionals formulate public policies and build good information platforms on health and well-being.

## RESUMO

**Objetivos:**

descrever o perfil dos idosos que acessam a internet para buscar informações sobre saúde e identificar os fatores que podem influenciar as decisões dos idosos sobre sua saúde com base nas informações coletadas online.

**Métodos:**

391 idosos responderam a um questionário online sobre hábitos e satisfação com informações sobre saúde coletadas na internet. Processamento dos dados envolveu regressão logística.

**Resultados:**

ensino superior reduz em 44% a probabilidade de idosos seguirem recomendações de saúde em sites da internet. Porém, atividades sociais e autopercepção de saúde aumentam a possibilidade de seguir as recomendações em 83% e 71%, respectivamente. A crença de que a internet promove hábitos saudáveis aumenta em 29,2 vezes a probabilidade de idosos seguirem os conselhos.

**Considerações Finais:**

conhecer o perfil dos idosos que utilizam a internet pode auxiliar os profissionais na formulação de políticas públicas e na construção de boas plataformas de informação sobre saúde e bem-estar.

## RESUMEN

**Objetivos:**

describir el perfil de los ancianos que acceden a internet para buscar información de salud e identificar los factores que pueden influir en las decisiones de los ancianos sobre su salud a partir de la información recopilada en línea.

**Métodos:**

391 ancianos respondieron a un cuestionario online sobre hábitos y satisfacción con la información de salud recogida en internet. El procesamiento de datos implicó regresión logística.

**Resultados:**

la educación superior reduce en un 44% la probabilidad de que un anciano siga las recomendaciones de salud en los sitios web. Sin embargo, las actividades sociales y la salud autopercibida aumentan la posibilidad de seguir las recomendaciones en un 83% y un 71%, respectivamente. La creencia de que internet promueve hábitos saludables aumenta 29,2 veces la probabilidad de que un anciano siga un consejo.

**Consideraciones Finales:**

conocer el perfil de los ancianos que utilizan internet puede ayudar a los profesionales a formular políticas públicas y construir buenas plataformas de información sobre salud y bienestar.

## INTRODUCTION

Increased life expectancy and world population aging have changed not only the demographic aspects but also the epidemiological structure. Increased chronic degenerative diseases in developed countries reflects this aging in different regions of the globe. It is no different in Brazil, as it is undergoing a rapid and intense aging process^(1)^. Projections indicate that, in 2050, older adults will make up 30% of the Brazilian population, and this is due to decreased birth rates and improved survival conditions. Moreover, recent medical advances and improved food supply have increased life expectancy worldwide^(2)^.

Recognizing the sociodemographic and epidemiological characteristics of this new population reality, such as habits and their relationship with information technologies, can help to implement actions and strategies that improve quality of life^(3)^.

The way aging affects older adults depends on their socioeconomic status. However, the participation of older adults in society is increasing, with greater engagement in family and community activities, which makes them agents that transform the social process and the provision of services^(4,5)^. Currently, older adults are more demanding consumers. The internet is a determining factor, as it offers faster and more dynamic access to information and leisure than in the past when information was transmitted by television or radio. The internet has recently changed people’s lives, providing online meetings, shopping, virtual travel, and even telemedicine consultations^(3)^.

Studies that analyze internet use by Brazilian older adults and its relationship with health and well-being information are relatively incipient, given the condition of digital exclusion still experienced by this population. A study involving 384 Brazilian older adults who used the social networks Facebook® and WhatsApp® aimed to establish the demographic profile of this population and the purpose of this use. The authors observed that health information is a potential focus of interest for these older adults when they browse the internet and digital social networks, since 65% of participants stated that they use it to resolve doubts about health care^(6)^.

However, this new reality may have disadvantages: it was found that internet access during the COVID-19 pandemic was also responsible for increased anxiety, stress, and depressive symptoms in older adults^(7)^. Furthermore, vulnerable individuals may use incomplete, incorrect, or unintelligible information from the internet to make decisions about their health or well-being^(8,9,10)^.

Older adults are a susceptible group to fake news online. American studies found that, during the 2016 US presidential campaign, people aged 65 and older were twice as likely to be exposed to fake news on Twitter® and seven times more likely to share fake news on Facebook® than young people^(11,12)^.

We are unaware of studies in the Brazilian population that have observed the profile of older adults who use the internet to obtain information about health and well-being and which characteristics of this population are relevant to decisions about health.

This knowledge can help recognize the sociodemographic and epidemiological characteristics of the Brazilian old adult population for creating public policies aimed at this population and considering the inclusion of older adults in the digital environment as a necessary form of access and positive influences for improving their lives.

## OBJECTIVES

To describe the profile of older adults who access the internet to search for health information and identify the factors that can influence older adults’ decisions about their health based on information collected online.

## METHODS

### Ethical aspects

The study was conducted following national and international ethics guidelines, and was approved by the Research Ethics Committee of *Instituto de Assistência Médica ao Servidor Público Estadual de São Paulo*, whose opinion is attached to this submission.

### Study design

The protocol of this study followed the STrengthening the Reporting of OBservational studies in Epidemiology (STROBE) recommendations. An exploratory, descriptive, cross-sectional quantitative design with binary Logistic Regression (LR) was used in this study. All participants agreed to participate in the research through the Informed Consent Form (ICF) available digitally.

### Sample and data collection

The convenience sampling technique was used since participation in the survey was voluntary. Data were collected over one year through an online collection in groups of older adults with Facebook® accounts.

For data collection, an announcement was made with an invitation to participate in the study on Facebook®, as this social network includes different groups. A website focused on older adults was the platform selected to host the form. Information on the authors of the study was also available on this website.

Participants provided written informed consent: when clicking on the survey form link, the ICF page appeared. The page with questions on the form could only be accessed if, at the end of reading the ICF, participants clicked on the option “I accept to participate in the research”.

An electronic form was created and structured in three parts. The first part was related to collecting socio-demographic identification data from study participants. The second part consisted of a guide for identifying lifestyle habits. The third was related to participants’ expectations regarding health information through the digital tool.

Electronic forms were made available and targeted to individuals aged 60 years or older, registered on social networks - who agreed to participate in the study by accepting the ICF between August 2017 and July 2018. The hosting page was the website “envelhecieagora.com,” where health and well-being issues are discussed focusing on older adults.

Individuals aged 60 years or older who access health and well-being websites and have the cognitive ability to complete a form on Google Forms® were included. Incomplete data and forms where respondents said they did not access health and well-being websites were excluded.

### Analysis of results, and statistics

The collected data were analyzed with the aid of SPSS22 software. First, descriptive statistics were used to analyze the variables. Then, Pearson’s chi-square test was used to test differences in the proportions of two or more variables, and p-values lower than 0.10 (10%) were considered significant.

As a tabulation technique for LR, the statement “Usually follows the guidelines related to health and well-being found on the internet” was chosen as dependent variable. Subsequently, independent variables were identified for the chi-square test and LR, such as age, gender, education, income, participation in social activities, leisure time activities, self-perceived health, if the respondent has any illness, reported disease, if the respondent is satisfied with the information available on the internet, if the information influences decisions and if the information promotes healthy habits.

The variables were selected through stepwise regression, considering levels of statistical significance of 10% for inclusion and exclusion of variables.

## RESULTS

The number of completed forms was 556, but only 391 individuals met the inclusion criteria and were selected to participate in the study.

Most respondents (76%) were between 60 and 70 years old, and the number of respondents over 71 years old was only 24%. Additionally, most respondents (93%) were women, and only 2% were men.

Respondents’ mean age was 65.7 ± 5.1. There was no difference in the age variable when we compared men and women (p=0.507).

Regarding marital status, most respondents (46%) declared to be married, 21%, widowed, 20%, divorced or separated, and only 13%, single.

Table 1 presents the population characteristics concerning education, income, and self-perceived health.

**Table 1:** Social characteristics and health and well-being of internet users who access health and wellness websites, São Paulo, São Paulo, Brazil, 2018

Variables	n	%
Education		
Elementary school	35	9%
High school	74	19%
Higher education	246	63%
Graduate education	36	9%
Total	**391**	**100%**
Income		
No income	15	4%
Up to 1 minimum wage	38	10%
2 to 3 minimum wages	119	30%
3 to 6 minimum wages	132	34%
More than 6 minimum wages	87	22%
Total	**391**	**100%**
Self-perceived health		
Excellent	87	22%
Good	212	54%
Regular	86	22%
Poor	4	1%
Does not know	2	1%
Total	**391**	**100%**
Has any illness		
Yes	270	69%
No	112	29%
Does not know	9	2%
Total	**391**	**100%**
Reported disease		
Depression	43	11%
Hypertension	152	39%
Diabetes	39	10%
Osteoporosis	45	12%
Neurological disorder	11	3%
Glaucoma	9	2%
Respiratory disease	15	4%
Heart disease	28	7%
Rheumatism and arthrosis	3	1%
Others	86	22%
Total	*431	-

* *More than one response per participant.*

Table 2 show the characteristics regarding life habits, satisfaction, attitudes about the information on health and well-being accessed on the internet.

**Table 2:** Distribution of characteristics related to life habits, satisfaction, belief, and influence of information in internet users who access health and well-being websites, São Paulo, São Paulo, Brazil, 2018

Variables	n	%
Leisure time activities		
Listens to the radio	70	18%
Socio-recreational activities	87	22%
Handicraft work	119	30%
Chat with friends	164	42%
Physical activity	198	51%
Reading	213	54%
Watches TV	230	59%
Browses the internet	340	87%
Others	86	23%
Total	*1507	-
Participation in social activities		
Recreational association	27	7%
Care association	24	6%
Sports association	16	4%
Labor union association	10	3%
Religious association	93	24%
Community association	25	6%
Does not participate	176	45%
Others	46	12%
Total	*417	-
Satisfaction with the information in the websites		
Yes	334	85%
No	57	15%
Total	391	100%
Believe that health and well-being websites promote healthy habits		
Yes	366	94%
No	25	6%
Total	391	100%
The information on websites influences their decisions		
Yes	283	72%
No	108	28%
Total	391	100%

For LR, the p-values of “yes” and “no” response scores were calculated for the statement “Usually follows the guidelines related to health and well-being on the internet.”

Variables of interest were selected step by step according to LR model optimization criteria, reducing variance and avoiding multicollinearity. Thus, only relevant variables for this study were selected (p=0.01).

The value obtained from the calculation of coefficient values represents the probability of following the guidelines related to health and well-being found on the internet. Table 3 shows the results of the admitted LR model.

**Table 3:** Significant independent variables in the Logistic Regression model, São Paulo, São Paulo, Brazil, 2018

Equation variables	B	SE	Wald	Df	Sig	Exp(B)
Education	-0.579	.376	2.366	1	0.124	0.561
Satisfied with the information	1.295	.375	11.935	1	0.001	3.652
Information influences	2.044	.301	46.013	1	0.000	7.21
Belives that websites promote healthy habits	3.374	.802	17.683	1	0.000	29.191
Participation in social activities	0.604	.296	4.159	1	0.041	1.830
Self-perceived health	0.535	.345	2.407	1	0.121	1.707
Constant	- 4.587	.977	22.051	1	0.000	.010

*B - Model coefficients for each explanatory variable; SE - standard error; Wald - Wald test, which is used to test the hypothesis of each coefficient of the regression model; Sig - statistical significance; Exp (B) indicates the exponential function of the estimated coefficients of each category of the model’s variables and indicates older adults’ chances of being influenced by information contained on the internet.*

## DISCUSSION

Based on socio-demographic characteristics and those related to internet use, significant variables in older adults’ choices to follow guidelines related to health and well-being can be determined.

In this study, the importance of LR as statistical analysis to assess the probability of a given behavior of a specific group was considered. It can be a helpful tool in studies aimed at identifying the probability of medication adherence, the risk of developing a disease, identification of the profile of older adults regarding adherence to vaccines, particularly during the COVID-19 pandemic, which generated vast flows of information, often conflicting, on the internet^(13)^.

The internet has changed people’s lives in several aspects of their routines. These new resources can be advantageous and contribute to improving older adults’ health. However, they can pose significant risks for vulnerable individuals who access the network and use incomplete or wrong information to make decisions about their health^(8,14,15)^.

The likelihood of internet engagement declines rapidly with age, and patterns of disengagement are more pronounced in older adults. This disengagement was detected in the present study, as older adults corresponded to the minor portion of those who completed the questionnaire. The most representative sample comprised individuals aged 60-70^(16,17)^.

Another critical factor was the number of female respondents. In addition to gender differences in internet use, there are different patterns of understanding and sources of health information. Men appear to be less interested in health-related issues. It is understood that they tend to be comparatively less willing and motivated to engage in these topics and generally seek health information online less frequently than women^(16,18,19,20)^.

It has been argued that gender differences can be caused by different reasons regarding the search for health information: women are more interested in health issues and emotional support, while men are more interested in informational support^(21)^.

According to studies^(18,19)^, women under 60 years old, with higher incomes and a high level of education are the users most likely to seek such health information on the internet.

Regarding educational level, individuals with higher education are more likely to seek health information in newspapers, magazines and on the internet^(22,23)^. A study published in 2008 reported that the effects of computer and internet use by older adults brings satisfaction, is useful and provides positive learning experiences. However, barriers such as physical and cognitive limitations, distrust and the time needed for learning can interfere with these individuals’ access to information^(17)^.

Therefore, it is possible to understand how older adults process new information, especially regarding learning technological skills, which for many are entirely new experiences and often unrelated to any prior knowledge^(23,24)^. Older adults are enthusiastic about using technologies that they consider practical. However, age, education, technical knowledge, and technological anxiety can affect their interest in new technologies, creating a technological divide. Thus, technologies that make this process more accessible and relevant to this population should be provided^(25)^.

A study conducted in 2004^(24)^ reveals that half of the adult American population is comfortable using the internet as a health information resource, and about a third of that population uses the internet to acquire health and well-being information. These findings are consistent with other relevant studies^(26,27)^.

Internet use has become essential in older adults’ routines. According to a US study, 82% of older adults aged over 65 years access the internet daily^(26)^.

Despite the growing use of the internet, there is little objective information about the impact of information available on the network regarding health promotion. Therefore, it is vital to understand the influence of such information, as this impact can be an obstacle or a facilitator in the relationship of older adults with health professionals^(28)^.

As reported in this study, health information acquisition on the internet by older adults can influence their health behaviors. Therefore, health professionals should seek to develop web-based interventions and programs to help older adults access accurate and appropriate health information^(27)^.

The characteristics of the older population influenced by information on the internet were observed, and binary LR was used to identify the characteristics that contribute to such influence.

In the investigation, the value obtained in calculating the coefficients represents the probability of acceptance of the guidelines related to health and well-being found on the internet. The coefficients interpretation and the impacts of the coefficients of independent variables on the odds ratio were observed, and the probability of an event occurring was determined. Moreover, it was found that a positive coefficient increases and a negative coefficient reduces the probability that the event occurs^(29)^.

Regarding self-perceived health, it is believed that, despite the challenges involved in general health status analysis, self-reported health is a generally effective measure of health status capable of identifying mental and social well-being aspects^(28,30,31)^.

Self-rated health is a reliable measure considering the aging process, including perception, cognition, and contextual factors^(28,30)^. Individuals who reported their health as poor have a higher risk of mortality than those who report their health as very good^(4,5,9,31)^. In our study, it was observed that most participants consider their health to be good or excellent, which is an essential part of the profile of internet users in this age group. These data may be associated with the fact that older adults who use the internet have a better cognitive level and less physical frailty^(32)^.

Thus, based on the results, after descriptive data analysis, LR analysis was applied to identify the profile of older adults influenced by the internet. Furthermore, the variables that contributed to participants’ decisions to follow the guidelines related to health and well-being were used.

The present study showed that the internet plays an essential role in access to information by older adults. These individuals use it to seek information about health and well-being, and based on such information, they can make decisions about their health. Therefore, health promotion programs and public policies targeted at older adults should consider access to information on the internet and the characteristics of the referred population.

Studies suggest that older adults have been using the internet to communicate with families and friends, in addition to video communication programs, online shopping, personal banking, blogging, gaming, or other activities. This tool is increasingly present in the lives of citizens of the most diverse age groups. Older adults use the internet to access health services, obtain health-related information or services, order medication and communicate with health professionals. Understanding how the internet influences these individuals’ choices can be essential to understanding how this information can impact older adults’ lives^(22,24,30,33)^.

The results of binary LR attempted to predict and explain, through the formula, which independent variables are correlated and influence the dependent variable, seeking to establish the probability that the variables contribute to the behavior shown in the sample. The coefficients estimated by the LR model showed the importance of each variable in the formula for identifying the profile of older adults influenced by information on the internet. The most important independent variables were information on websites, and health and well-being influence the promotion of healthy habits. The results can be interpreted by the probability of measuring how an older adult with a specific profile behaves regarding their health and well-being.

This study showed the impact of the variables studied on old adults’ decisions about health based on the information on websites: a higher level of education reduces the probability of an older adult following websites’ guidelines by 44%. As expected, the belief that these websites promote health and well-being increases the chances of following the guidelines by almost 30 times. Notably, participation in social activities and self-perceived health increased by 83% and 71% the chances of an older adult following the guidelines found on the internet.

The profile of older adults who use the internet to obtain information about health and well-being observed in this study can serve as a basis for creating policies or incentives to build information platforms about health and well-being that expect and are competent for this population.

### Study limitations

The study was limited by the sample consisting mostly of women.

### Contributions to nursing, health or public policies

Nursing professionals must monitor the population’s ways of accessing health information. Understanding how older adults use the internet is essential to improving access to pertinent information about health and well-being. This understanding allows us to adapt data in an understandable and valuable way to this group, considering their needs, technological skills, and preferences. By understanding connected older adults’ profiles, these professionals can develop strategies to promote health and facilitate access to online services, such as creating user-friendly platforms with reliable information about prevention, medical conditions, and self-care.

## CONCLUSIONS

Older adults who use the internet to obtain information on health and well-being are usually women, with higher education, an income of more than two minimum wages, married, and reporting health problems, the most common being hypertension. These older adults tend to browse the internet daily during leisure time, and the information on websites tends to influence decision-making regarding health.

Education has a negative impact on the influence of internet information on health-related decision-making. However, satisfaction with internet information, participation in social activities, self-perceived health, and the belief that the internet promotes healthy habits are variables that positively impact decisions related to health and well-being.

## Supplementary Material

0034-7167-reben-77-01-e20230321-suppl01

## References

[1] Ramón-Jerónimo MA, Peral-Peral B, Arenas-Gaitán J. (2013). Elderly persons and internet use. Soc Sci Comput Rev.

[2] Lima-Costa MF, Matos DL, Camargos VP, Macinko J. (2011). Tendências em dez anos das condições de saúde de idosos brasileiros: evidências da Pesquisa Nacional por Amostra de Domicílios (1998, 2003, 2008). Cien Saude Colet.

[3] Leavengood LB. (2001). Older people and Internet use. Generations J Am Soc Aging [Internet].

[4] Beard JR, Officer A, Carvalho IA, Sadana R, Pot AM, Michel JP, (2016). The World report on ageing and health: a policy framework for healthy ageing. Lancet.

[5] Dontsov VI, Krut’ko VN, Mitrohin OV. (2020). Decrease in Human Aging Rate Since the Middle of the 20th Century. Dokl Biol Sci.

[6] Diniz JL, Moreira ACA (2020). Inclusão digital e o uso da internet pela pessoa idosa no Brasil: estudo transversal. Rev Bras Enferm.

[7] Fhon JRS, Püschel VAA, Cavalcante RB, Cruz FV, Gonçalves LN, Li W, (2022). Infodemic of covid-19 and repercussions on the mental health of the elderly from São Paulo. Rev Esc Enferm USP.

[8] Oermann MH. (2003). Using health web sites for patient education. J Wound Ostomy Continence Nurs.

[9] Shapira N, Barak A, Gal I. (2007). Promoting older adults’ well-being through internet training and use. Aging Ment Health.

[10] Koch-Weser S, Bradshaw YS, Gualtieri L, Gallagher SS. (2010). The Internet as a health information source: findings from the 2007 Health Information National Trends Survey and implications for health communication. J Health Commun.

[11] Grinberg N, Joseph K, Friedland L, Swire-Thompson B, Lazer D. (2019). Fake news on Twitter during the 2016 U.S. presidential election. Science.

[12] Guess A, Nagler J, Tucker J. (2019). Less than you think: prevalence and predictors of fake news dissemination on Facebook. Sci Adv.

[13] Kupek E. (2021). Low COVID-19 vaccination coverage and high COVID-19 mortality rates in Brazilian elderly. Rev Bras Epidemiol.

[14] Arai H, Ouchi Y, Yokode M, Ito H, Uematsu H (2012). Toward the realization of a better aged society: messages from gerontology and geriatrics. Geriatr Gerontol Int.

[15] Cotten SR, Ford G, Ford S, Hale TM. (2014). Internet use and depression among retired older adults in the United States: a longitudinal analysis. J Gerontol B Psychol Sci Soc Sci.

[16] Gheorghiu B, Hagens S. (2017). Use and Maturity of Electronic Patient Portals. Stud Health Technol Inform [Internet].

[17] Gatto SL, Tak SH. (2008). Computer, internet, and e-mail use among older adults: benefits and barriers. Education Gerontol.

[18] Kimbrough AM, Guadagno RE, Muscanell NL, Dill J. (2013). Gender differences in mediated communication: women connect more than do men. Comput Human Behav.

[19] Rowley J, Johnson F, Sbaffi L. (2017). Gender as an influencer of online health information‐seeking and evaluation behavior. J Assoc Informat Sci Technol.

[20] Carvalho ML, Barbosa CNS, Bezerra V P, Santos AMRD, Silva CRDT, Brito CMS, (2019). Health situation in the perception of elderly widows assisted by primary health care. Rev Bras Enferm.

[21] Fischer SH, David D, Crotty BH, Dierks M, Safran C. (2014). Acceptance and use of health information technology by community-dwelling elders. Int J Med Inform.

[22] Marin-Torres V, Valverde Aliaga J, Sánchez Miró I, Del Castillo Vicente MIS, Polentinos-Castro E, Garrido Barral A. (2013). Internet as an information source for health in primary care patients and its influence on the physician-patient relationship. Aten Primaria.

[23] Huber L, Watson C. (2014). Technology: education and training needs of older adults. Education Gerontol.

[24] Fox S. (2004). Older Americans and the internet. Pew Internet and American Life Project [Internet].

[25] Xavier AJ, d’Orsi E, Wardle J, Demakakos P, Smith SG, von Wagner C. (2013). Internet use and cancer-preventive behaviors in older adults: findings from a longitudinal cohort study. Cancer Epidemiol Biomarkers Prev.

[26] Zickuhr K, Madden M. (2012). Older Adults and Internet Use. Pew Res Center’s Internet Am Life Proj [Internet].

[27] Hunsaker A, Hargittai E. (2018). A review of Internet use among older adults. New Media Soc.

[28] Jin K, Simpkins JW, Ji X, Leis M, Stambler I. (2014). The critical need to promote research of aging and aging-related diseases to improve health and longevity of the elderly population. Aging Dis.

[29] Melo LA, Ferreira LMBM, Santos MM, Lima KC. (2017). Socioeconomic, regional and demographic factors related to population ageing. Rev Bras Geriatr Gerontol.

[30] Marôco J. (2018). Análise Estatística com o SPSS Statistics.

[31] Silva AF, Cancela JM, Mollinedo I, Camões M, Bezerra P. (2021). The relationship between health perception and health predictors among the elderly across European Countries. Int J Environ Res Public Health.

[32] Mello BH, Lenardt MH, Moraes DC, Setoguchi LS, Seima MD, Betiolli SE. (2021). Cognitive impairment and physical frailty in older adults in secondary health care. Rev Esc Enferm USP.

[33] Chang J, McAllister C, McCaslin R. (2015). Correlates of, and barriers to, Internet use among older adults. J Gerontol Soc Work.

