# Supplementary material for: The influence of the internet on choices about older adults’ health and well-being
Source: Rev Bras Enferm. 2024 May 13;77(1):e20230321. doi: 10.1590/0034-7167-2023-0321 (PMC11095947; doi:10.1590/0034-7167-2023-0321)
Supplement: 0034-7167-reben-77-01-e20230321-suppl01 [file 0034-7167-reben-77-01-e20230321-suppl01.pdf]

Date	A-1. Qual sua idade? Em anos completos:	A-2. Quais as iniciais de seu nome:	A-4. Sexo:	A-5. Estado Civil atual:	A-6. Escolaridade		A-8. Atualmente o sr(a) mora:			A-11. Caso esteja aposentado(a), com que idade se aposentou em anos completos:	A-12. Qual o motivo de sua aposentadoria?	A-13. Qual sua renda mensal? Reais:	A-13. Qual sua principal fonte de renda:		B- 01 Faz uso de cigarros e/ou charutos, cachimbo?	B- 02. Faz uso de medicamentos:	B- 03. Se respondeu sim na pergunta anterior que tipo:	
2017-05-16	62	JBS	Feminino	Casado(a)	Ensino Superior			Outros	Sim	Entre 40 e 52 anos			aposentadoria	divide responsabilidades		Sim		
2017-10-13	64	ACB	Feminino	Solteiro(a)	Ensino Superior		com filho(s)		Sim	Entre 40 e 52 anos			aposentadoria			Sim		1,2
2017-12-03	61	MGDR	Feminino	Solteiro(a)	Ensino Superior			Outros	Sim	Entre 53 a 60 anos			aposentadoria	divide responsabilidades		Sim		1,5
2018-02-11	77	VS	Feminino				sozinho		Sim	Entre 53 a 60 anos	idade		aposentadoria e outros			Sim		1,2
2018-02-19	65	EC	Feminino	Divorciado(a)			sozinho	Do lar	Sim	Entre 53 a 60 anos	idade					Sim		1,7
2018-02-20	73	DBS	Feminino	Casado(a)	Ensino Superior			Professor(a)	Sim	Entre 40 e 52 anos			aposentadoria	divide responsabilidades		Sim		1,5
2018-02-20	62	RSV	Feminino	Casado(a)	Ensino Superior			Outros	Sim	Entre 40 e 52 anos			aposentadoria	divide responsabilidades		Sim		1,5
2018-02-21	73	MRRP	Feminino	Casado(a)	Ensino Superior			Professor(a)	Sim	Entre 53 a 60 anos			aposentadoria	divide responsabilidades		Sim		1,2
2018-02-24	71	I C S	Feminino	Divorciado(a)	Ensino Superior		sozinho	Professor(a)	Sim	Acima de 60 anos			aposentadoria			Sim		1,2
2018-03-07	60	VAX	Feminino	Divorciado(a)	Ensino Superior		com filho(s)	Outros								Sim		1,2
2018-03-11	70	GAS	Feminino	Solteiro(a)	Ensino Superior	cedida		Professor(a)	Sim	Entre 53 a 60 anos			aposentadoria e outros			Sim		1,5
2017-04-30	74	NPB	Feminino				sozinho		Sim	Acima de 60 anos	idade					Sim		
2017-05-08	60	d f	Feminino	Divorciado(a)	Ensino Superior	alugada	com filho(s)	Professor(a)	Sim	Entre 53 a 60 anos						Sim		
2018-02-25	62	OSGS	Masculino	Casado(a)				Outros	Sim	Entre 53 a 60 anos			aposentadoria e outros			Sim		
2017-11-16	69	R F R	Feminino	Casado(a)	Ensino Superior	alugada		Professor(a)	Sim	Entre 53 a 60 anos			aposentadoria		sim	Sim		
2018-03-25	63	MCTR	Feminino	Divorciado(a)			sozinho	Do lar	Sim	Entre 40 e 52 anos	aposentadoria especial					Sim		2,4,7,8
2018-02-16	73	M z	Feminino				sozinho	Do lar	Sim	Entre 53 a 60 anos	idade		aposentadoria e outros		sim	Sim		2,4,7,8
2018-03-01	67	JB	Feminino	Solteiro(a)	Ensino Superior			Outros		Entre 53 a 60 anos			aposentadoria e outros			Sim	7- Dor, 12- Outros	7,8,12
2018-02-10	67	M V	Feminino		Ensino Superior		com filho(s)	Professor(a)	Sim	Entre 53 a 60 anos			aposentadoria			Sim		2,4,8,9
2017-10-13	65	Hpa	Feminino	Casado(a)			com companheiros(a), filho(s) e neto(s)						outros	divide responsabilidades		Sim	12- Outros	
2017-11-10	62	MAS	Feminino	Casado(a)			com companheiro(a) e filhos	Outros						divide responsabilidades		Sim	12- Outros	
2017-11-21	60	Mc	Feminino	Solteiro(a)	Ensino Superior		com netos	Professor(a)								Sim	12- Outros	
2017-05-06	68	E.S.	Feminino				sozinho	Do lar	Sim	Acima de 60 anos	idade		aposentadoria			Sim	12- Outros	
2017-12-09	72	CLFS	Feminino	Casado(a)	Ensino Superior			Professor(a)	Sim	Entre 40 e 52 anos			aposentadoria e outros	divide responsabilidades		Sim		2,9,12
2017-10-02	76	VS	Feminino				sozinho		Sim	Acima de 60 anos	idade		aposentadoria e outros			Sim		1,2,10
2017-05-20	62	J.N.S.L.	Feminino	Casado(a)	Ensino Superior			Professor(a)	Sim	Entre 53 a 60 anos	aposentadoria especial		aposentadoria			Sim		2,4,8
2017-10-13	70	M.M.P.	Feminino	Casado(a)				Outros	Sim	Acima de 60 anos	idade		aposentadoria	divide responsabilidades		Sim		2,8
2017-11-28	62	JBCC	Masculino	Casado(a)	Ensino Superior			Outros	Sim	Entre 53 a 60 anos			aposentadoria			Sim		2,8
2018-02-25	67	M T	Feminino	Divorciado(a)		alugada	com parentes outros	Do lar	Sim	Acima de 60 anos	idade		outros	divide responsabilidades		Sim		2,8
2018-03-07	69	E A R	Feminino	Casado(a)	Ensino Superior			Outros	Sim	Acima de 60 anos	idade		aposentadoria			Sim		2,8
2018-03-08	60	RSAP	Feminino	Casado(a)			com companheiro(a) e filhos	Outros	Sim	Entre 53 a 60 anos			aposentadoria	divide responsabilidades		Sim		2,8
2018-03-11	60	SH	Feminino	Casado(a)	Ensino Superior		com companheiro(a) e filhos	Do lar	Sim	Entre 53 a 60 anos	idade		aposentadoria			Sim		2,8
2018-02-14	63	LO	Feminino	Solteiro(a)		outros	com parentes outros	Professor(a)	Sim	Entre 53 a 60 anos	idade		aposentadoria	divide responsabilidades		Sim		2,4,6,7

2017-05-07	72M,,j,,c,,s	Feminino	Divorciado(a)		outros	com parentes outros	Do lar	Sim	Acima de 60 anos	idade	aposentadoria	divide responsabilidades		Sim		2,6,7
2017-10-22	69LF	Feminino	Divorciado(a)	Ensino Superior		com filho(s)	Professor(a)	Sim	Entre 40 e 52 anos		aposentadoria	divide responsabilidades		Sim		2,4,7,
2017-12-09	65SMP	Feminino	Divorciado(a)			com filho(s)	Outros	Sim	Entre 53 a 60 anos		aposentadoria			Sim		2,4,7
2018-03-20	61CLL	Feminino	Casado(a)				Profissional liberal	Sim	Entre 53 a 60 anos	idade	aposentadoria	divide responsabilidades		Sim		2,4,7
2017-05-15	67Z N M	Feminino	Casado(a)				Professor(a)	Sim	Acima de 60 anos		aposentadoria	divide responsabilidades		Sim		2,7,12
2018-03-04	70SMG	Feminino	Solteiro(a)	Ensino Superior		com parentes outros	Financeiro/ Administrativo	Sim	Entre 53 a 60 anos	idade	outros	divide responsabilidades		Sim		2,7,12
2018-02-27	61L H	Feminino	Casado(a)	Ensino Superior			Do lar							Sim		2,7
2018-02-14	68MLD	Feminino	Solteiro(a)	Ensino Superior		sozinho	Outros	Sim	Entre 40 e 52 anos		aposentadoria			Sim	3- Diabetes, 7- Dor, 8- Reumatismo	2,3,7,
2017-05-19	61RRE	Feminino	Casado(a)				Do lar							Sim		2,3,4,
2017-05-08	63Mcvo	Feminino	Casado(a)	Ensino Superior			Professor(a)	Sim	Entre 53 a 60 anos		aposentadoria	divide responsabilidades		Sim		2,3,4,
2017-05-22	70CRM	Feminino	Divorciado(a)	Ensino Superior		sozinho		Sim	Entre 53 a 60 anos		outros			Sim		2,3,4
2017-10-13	70Es	Feminino	Solteiro(a)	Ensino Superior		sozinho	Financeiro/ Administrativo	Sim	Entre 53 a 60 anos	idade	aposentadoria			Sim		2,3,5,
2017-11-29	69LAS	Feminino		Ensino Superior		sozinho	Professor(a)	Sim	Entre 53 a 60 anos		aposentadoria			Sim		2,5,6
2017-11-13	72M A A	Feminino			outros	com filho(s)	Do lar	Sim	Entre 53 a 60 anos	idade	aposentadoria			Sim		2,3,4,
2018-02-09	65Shl	Feminino	Divorciado(a)	Ensino Superior		com filho(s)	Professor(a)	Sim	Entre 53 a 60 anos		aposentadoria			Sim	3- Diabetes, 5- Colesterol12- Outros	2,3,5,
2018-02-13	80TJcfs	Feminino	Casado(a)				Professor(a)	Sim	Acima de 60 anos	idade	aposentadoria	divide responsabilidades		Sim	3- Diabetes, 5- Colesterol	2,3,5,
2018-03-13	67SMF	Feminino	Divorciado(a)		alugada	sozinho	Do lar	Sim	Entre 53 a 60 anos	idade	aposentadoria e outros			Sim		2,3,4,
2018-02-18	61JA	Masculino	Casado(a)				Outros	Sim	Entre 53 a 60 anos		aposentadoria			Sim	3- Diabetes, 5- Colesterol, 12- Outros	2,3,5,
2018-03-13	60R.	Feminino	Solteiro(a)		outros		Outros							Sim	3- Diabetes, 5- Colesterol, 12- Outros	2,3,5,
2017-10-19	76mlbxs	Feminino	Casado(a)				Do lar				outros			Sim	3- Diabetes, 5- Colesterol	2,3,5
2017-11-18	61MFMG	Feminino		Ensino Superior		com filho(s)	Professor(a)	Sim	Entre 53 a 60 anos		aposentadoria e outros			Sim		2,5
2018-02-14	63M.A.N.	Feminino	Solteiro(a)			sozinho	Outros	Sim	Entre 40 e 52 anos		aposentadoria		sim	Sim	3- Diabetes, 5- Colesterol	2,3,5
2018-02-17	70ESM	Feminino		Ensino Superior		com parentes outros		Sim	Entre 40 e 52 anos		aposentadoria	divide responsabilidades		Sim		
2018-02-20	60H L L C R	Feminino	Casado(a)		cedida	com parentes outros	Do lar							Sim	3- Diabetes, 5- Colesterol	2,3,5
2018-03-02	65TMM	Feminino	Casado(a)	Ensino Superior			Outros	Sim	Entre 40 e 52 anos	aposentadoria especial	aposentadoria	divide responsabilidades		Sim	3- Diabetes, 5- Colesterol	2,3,5
2018-03-18	69LAS	Feminino		Ensino Superior		sozinho	Professor(a)	Sim	Entre 53 a 60 anos					Sim	3- Diabetes, 5- Colesterol	2,3,5
2017-10-05	67Nmm	Feminino	Casado(a)					Sim	Entre 40 e 52 anos		aposentadoria	divide responsabilidades		Sim		2,5
2018-02-10	63Mfla	Feminino	Casado(a)			com filho(s)	Do lar	Sim	Entre 40 e 52 anos		aposentadoria			Sim	3- Diabetes	1,2,3
2017-09-27	64J RC	Feminino	Divorciado(a)		alugada	com parentes outros	Do lar							Sim		2,3
2017-11-12	76G. M	Masculino	Casado(a)	Ensino Superior			Engenheiro(a)	Sim	Entre 40 e 52 anos	aposentadoria especial	aposentadoria			Sim		2,3,5
2018-02-20	68MTMS	Feminino	Casado(a)	Ensino Superior			Advogado(a)							Sim		2,3
2018-02-20	74APC	Feminino	Casado(a)	Ensino Superior			Financeiro/ Administrativo	Sim	Entre 40 e 52 anos		aposentadoria	divide responsabilidades		Sim		2,3
2018-03-07	66GPN	Masculino	Divorciado(a)		alugada	sozinho	Outros						sim	Sim		2,3
2018-03-11	70M	Feminino	Casado(a)	Ensino Superior			Professor(a)	Sim	Entre 40 e 52 anos		aposentadoria			Sim		2,3,5
2017-10-03	81Y A C K	Feminino		Ensino Superior		sozinho	Professor(a)	Sim	Entre 53 a 60 anos		aposentadoria e outros			Sim		2,3
2017-10-09	67J C G S	Masculino	Casado(a)	Ensino Superior		com companheiro(a) e filhos		Sim	Entre 40 e 52 anos		aposentadoria			Sim		2,3
2018-03-07	60BLSF	Feminino	Casado(a)	Ensino Superior	alugada	com companheiro(a) e filhos	Outros	Sim	Entre 40 e 52 anos		aposentadoria	divide responsabilidades		Sim		2,4,9

2017-10-23	67	GL	Feminino	Solteiro(a)			sozinho	Outros	Sim	Acima de 60 anos		aposentadoria			Sim	2,4,12
2017-11-12	60	LR	Feminino	Casado(a)			com companheiro(a) e filhos	Do lar				outros			Sim	2,4,12
2017-12-07	87	Tp	Feminino			alugada	sozinho	Outros	Sim	Entre 40 e 52 anos		aposentadoria e outros			Sim	2,4,12
2017-05-19	65	Ls	Feminino	Divorciado(a)	Ensino Superior			Outros	Sim	Entre 40 e 52 anos		aposentadoria		sim	Sim	2,4
2017-10-21	67	GFSN	Feminino			alugada	com parentes outros	Profissional liberal	Sim	Acima de 60 anos	idade				Sim	2,4
2017-11-15	67	J R L	Masculino	Casado(a)	Ensino Superior		com companheiro(a) e filhos	Outros				outros		sim	Sim	2,4
2018-02-18	76	A.A.	Feminino	Divorciado(a)	Ensino Superior		com filho(s)	Professor(a)	Sim	Acima de 60 anos	idade	aposentadoria	divide responsabilidades	sim	Sim	2,4
2018-03-12	64	MMCVT	Feminino	Casado(a)	Ensino Superior	alugada		Do lar	Sim	Acima de 60 anos	idade	aposentadoria	divide responsabilidades		Sim	2,4
2018-03-16	71	I M P M	Feminino	Casado(a)	Ensino Superior			Professor(a)	Sim	Entre 40 e 52 anos		aposentadoria	divide responsabilidades		Sim	2,4
2017-09-27	61	GGA	Feminino	Solteiro(a)	Ensino Superior	cedida	sozinho	Professor(a)							Sim	2,4
2017-10-01	61	CLL	Feminino	Casado(a)				Profissional liberal	Sim	Entre 53 a 60 anos	idade	aposentadoria e outros	divide responsabilidades		Sim	2,4
2017-10-01	78	M A S A	Feminino				sozinho	Professor(a)	Sim	Entre 53 a 60 anos		aposentadoria	divide responsabilidades		Sim	2,4
2017-10-22	69	R R	Feminino	Casado(a)	Ensino Superior		com companheiro(a) e filhos	Professor(a)	Sim	Acima de 60 anos		aposentadoria	divide responsabilidades		Sim	2,5,9
2017-11-14	76	M.A.O.N.	Feminino	Casado(a)	Ensino Superior	alugada		Outros	Sim	Entre 40 e 52 anos		aposentadoria	divide responsabilidades		Sim	2,5,7,8
2018-03-01	64	M G	Feminino	Casado(a)	Ensino Superior	alugada	sozinho	Professor(a)	Sim	Entre 40 e 52 anos		aposentadoria			Sim	2,5,7,8
2017-10-03	67	E	Feminino	Casado(a)	Ensino Superior				Sim	Acima de 60 anos	idade	aposentadoria	divide responsabilidades		Sim	2,5,7,8
2017-11-15	72	C S M	Feminino	Casado(a)	Ensino Superior		com companheiro(a) e filhos	Professor(a)	Sim	Entre 53 a 60 anos		aposentadoria	divide responsabilidades		Sim	2,5,8
2017-11-12	65	A	Feminino	Casado(a)	Ensino Superior				Sim	Acima de 60 anos		aposentadoria	divide responsabilidades		Sim	2,5,6
2017-11-19	73	ICFP	Feminino	Casado(a)	Ensino Superior			Professor(a)	Sim	Acima de 60 anos		aposentadoria			Sim	2,5,6
2018-02-15	61	E A F A	Feminino	Casado(a)	Ensino Superior			Professor(a)							Sim	2,5,6
2017-10-04	69	I N P	Feminino	Casado(a)	Ensino Superior				Sim	Entre 53 a 60 anos	idade	aposentadoria e outros	divide responsabilidades		Sim	2,4,5,7
2017-10-13	65	MMA	Feminino	Casado(a)	Ensino Superior			Outros	Sim	Entre 40 e 52 anos		aposentadoria			Sim	2,5,7
2018-03-04	69	NMR	Feminino	Casado(a)				Outros	Sim	Entre 53 a 60 anos		aposentadoria	divide responsabilidades		Sim	2,5,7
2018-03-11	61	MRDC	Feminino	Solteiro(a)	Ensino Superior		com filho(s)	Professor(a)					divide responsabilidades		Sim	2,5,7
2017-11-18	75	De	Feminino	Casado(a)				Do lar				outros	divide responsabilidades		Sim	2,4,5,1
2017-12-02	75	D L B B	Feminino	Casado(a)				Do lar				outros			Sim	2,4,5,1
2018-02-10	67	DNM	Feminino	Casado(a)	Ensino Superior			Profissional liberal	Sim	Entre 40 e 52 anos		aposentadoria	divide responsabilidades	sim	Sim	2,4,5,1
2017-05-15	71	D.Ldel C. I.A.	Feminino	Divorciado(a)	Ensino Superior		sozinho	Profissional liberal	Sim	Acima de 60 anos	idade	aposentadoria			Sim	2,4,5
2017-10-21	74	MC	Feminino		Ensino Superior	cedida	com parentes outros	Outros	Sim	Acima de 60 anos	idade	aposentadoria			Sim	2,4,5
2017-10-21	60	RNG	Feminino	Divorciado(a)	Ensino Superior	alugada	sozinho		Sim	Entre 53 a 60 anos		aposentadoria e outros	divide responsabilidades	sim	Sim	2,4,5
2017-10-22	62	BP	Feminino			alugada	com parentes outros	Outros				outros	divide responsabilidades		Sim	2,4,5
2017-11-14	74	M S M L	Feminino		Ensino Superior		com filho(s)	Outros	Sim	Entre 40 e 52 anos					Sim	2,4,5
2017-11-16	60	MRB	Feminino	Divorciado(a)	Ensino Superior	alugada	com filho(s)	Outros	Sim	Entre 53 a 60 anos		aposentadoria			Sim	2,4,5
2018-03-07	73	LMZH	Feminino		Ensino Superior		sozinho	Professor(a)	Sim	Entre 40 e 52 anos		aposentadoria e outros			Sim	2,4,5
2018-03-08	68	G.O.G.	Feminino	Solteiro(a)	Ensino Superior		sozinho	Profissional liberal	Sim	Acima de 60 anos		aposentadoria			Sim	2,4,5
2017-09-26	78	LFPF	Feminino	Casado(a)				Engenheiro(a)	Sim	Entre 40 e 52 anos		aposentadoria	divide responsabilidades		Sim	1,4,5
2017-10-01	63	A A C	Feminino	Casado(a)				Outros	Sim	Entre 53 a 60 anos		aposentadoria e outros	divide responsabilidades		Sim	2,5,12

2017-10-15	60Jslm	Feminino	Casado(a)	Ensino Superior				Sim	Entre 53 a 60 anos		outros	divide responsabilidades		Sim		2,5,12
2017-11-12	61MLC	Feminino	Casado(a)			com companheiro(a) e filhos	Professor(a)	Sim	Entre 40 e 52 anos	aposentadoria especial	aposentadoria	divide responsabilidades		Sim		2,5,12
2017-11-13	60LCGK	Feminino	Divorciado(a)	Ensino Superior		com filho(s)	Outros	Sim	Entre 40 e 52 anos		aposentadoria			Sim		2,5,12
2018-02-13	61MLM	Feminino	Solteiro(a)	Ensino Superior		com filho(s)	Outros	Sim	Entre 53 a 60 anos		aposentadoria			Sim		2,5,12
2018-02-15	73Los	Feminino	Casado(a)	Ensino Superior			Professor(a)	Sim	Entre 53 a 60 anos		aposentadoria	divide responsabilidades		Sim		2,5,12
2018-02-15	66RQA	Feminino	Divorciado(a)	Ensino Superior		sozinho	Professor(a)		Entre 40 e 52 anos					Sim		2,5,12
2018-03-13	80GVF	Masculino	Casado(a)	Ensino Superior			Financeiro/ Administrativo	Sim	Entre 40 e 52 anos		aposentadoria	divide responsabilidades		Sim		2,5,12
2018-03-22	64EK	Feminino	Divorciado(a)			sozinho	Do lar				outros			Sim		2,5,12
2017-05-19	64FMMB	Feminino	Casado(a)	Ensino Superior		com companheiro(a) e filhos	Outros	Sim	Entre 53 a 60 anos		aposentadoria			Sim		2,5
2017-09-27	65A	Feminino	Casado(a)	Ensino Superior				Sim	Acima de 60 anos		aposentadoria	divide responsabilidades		Sim		2,5
2017-10-12	60E M	Feminino	Casado(a)			com companheiro(a) e filhos	Outros				outros	divide responsabilidades		Sim	12- Outros	
2017-10-21	66DVGF	Feminino			alugada	sozinho	Professor(a)	Sim	Entre 53 a 60 anos					Sim		2,5
2017-11-11	70MHSK	Feminino		Ensino Superior		sozinho	Outros	Sim	Entre 40 e 52 anos		aposentadoria			Sim		2,5
2017-11-12	66SMTSS	Feminino		Ensino Superior		sozinho	Professor(a)	Sim	Entre 53 a 60 anos		aposentadoria e outros			Sim		2,5
2017-11-12	60MCMDS	Feminino	Casado(a)	Ensino Superior			Professor(a)	Sim	Entre 53 a 60 anos		aposentadoria	divide responsabilidades		Sim		2,5
2017-11-15	66CSS	Feminino	Casado(a)				Do lar				outros			Sim		2,5
2017-11-19	76MA	Feminino		Ensino Superior		sozinho	Profissional liberal	Sim	Acima de 60 anos		aposentadoria e outros			Sim		2,5
2017-11-28	65SMPS	Feminino	Casado(a)				Profissional liberal	Sim	Entre 53 a 60 anos	idade	aposentadoria e outros			Sim		2,5
2017-12-01	62M F A A	Feminino			alugada	com parentes outros	Professor(a)	Sim	Entre 53 a 60 anos		aposentadoria e outros			Sim		2,5
2018-02-09	64mbk	Feminino	Solteiro(a)			com netos	Outros	Sim	Entre 40 e 52 anos		aposentadoria	divide responsabilidades		Sim		2,5
2018-02-11	64Me	Feminino	Casado(a)	Ensino Superior		com companheiro(a) e filhos	Outros	Sim	Entre 53 a 60 anos		aposentadoria	divide responsabilidades	sim	Sim		2,5
2018-02-11	68JPN	Feminino	Solteiro(a)	Ensino Superior	alugada	sozinho	Financeiro/ Administrativo	Sim	Entre 40 e 52 anos		aposentadoria			Sim		2,5
2018-02-13	64FMMB	Feminino	Casado(a)	Ensino Superior		com companheiro(a) e filhos	Outros	Sim	Acima de 60 anos		aposentadoria			Sim		2,5
2018-02-20	76Mlbx	Feminino	Casado(a)				Do lar							Sim		2,5
2018-02-21	65MMF	Feminino	Divorciado(a)	Ensino Superior		com filho(s)	Professor(a)	Sim	Entre 40 e 52 anos		aposentadoria			Sim		2,5
2018-03-04	61gc	Feminino	Casado(a)	Ensino Superior			Professor(a)	Sim	Entre 53 a 60 anos		aposentadoria			Sim		2,5
2018-03-23	69F.C.	Feminino	Solteiro(a)	Ensino Superior		sozinho	Advogado(a)	Sim	Acima de 60 anos	idade	aposentadoria			Sim		2,5
2017-10-01	60IM	Feminino	Divorciado(a)	Ensino Superior		com filho(s)	Engenheiro(a)	Sim	Entre 53 a 60 anos			divide responsabilidades		Sim		2,5
2017-10-02	61NMMF	Feminino	Casado(a)	Ensino Superior			Professor(a)	Sim	Entre 53 a 60 anos		aposentadoria	divide responsabilidades		Sim		2,5
2017-10-05	67SS	Feminino	Divorciado(a)	Ensino Superior		com filho(s)		Sim	Acima de 60 anos	idade	aposentadoria			Sim		2,5
2017-10-13	63GFM	Feminino	Solteiro(a)	Ensino Superior		sozinho	Outros	Sim	Acima de 60 anos		aposentadoria			Sim		2,12
2017-10-21	70MG	Feminino	Divorciado(a)	Ensino Superior		sozinho	Professor(a)	Sim	Entre 53 a 60 anos		aposentadoria			Sim		2,12
2017-11-14	71MECS	Feminino	Solteiro(a)			sozinho		Sim	Acima de 60 anos		aposentadoria			Sim		2,12
2017-11-20	66Ict	Feminino	Divorciado(a)	Ensino Superior			Outros	Sim	Entre 53 a 60 anos		aposentadoria			Sim		2,12
2018-02-13	77EFM	Feminino				sozinho	Do lar	Sim	Acima de 60 anos	idade				Sim		2,12
2018-02-13	61Acv	Feminino	Divorciado(a)		alugada	com parentes outros		Sim	Entre 40 e 52 anos					Sim		2,12
2018-03-03	73N R D	Feminino	Casado(a)				Outros				outros			Sim		2,12
2018-03-10	73NRD	Feminino	Casado(a)				Profissional liberal							Sim		2,12
2018-03-12	66M.I.B	Feminino				com parentes outros	Do lar	Sim	Entre 53 a 60 anos	idade		divide responsabilidades	sim	Sim		2,12
2018-03-18	69JMGC	Feminino	Divorciado(a)			com parentes outros		Sim	Acima de 60 anos					Sim		2,12
2018-03-21	75A A	Feminino	Casado(a)	Ensino Superior			Outros	Sim	Entre 40 e 52 anos		aposentadoria e outros	divide responsabilidades		Sim		2,12

2017-09-25	64Tof	Feminino	Solteiro(a)	Ensino Superior		com filho(s)		Sim	Entre 40 e 52 anos		aposentadoria			Sim
2017-05-11	63obmf	Masculino	Casado(a)			com companheiros(a), filho(s) e neto(s)	Outros					divide responsabilidades		Sim
2017-05-14	66EAN	Masculino	Solteiro(a)		cedida	sozinho		Sim	Acima de 60 anos	idade	aposentadoria			Sim
2017-05-20	64A C	Feminino	Divorciado(a)	Ensino Superior	alugada	sozinho								Sim
2017-05-21	62ARPO	Feminino	Casado(a)				Professor(a)	Sim	Entre 40 e 52 anos		aposentadoria			Sim
2017-09-30	62H L	Feminino	Casado(a)	Ensino Superior		com parentes outros		Sim	Entre 53 a 60 anos		aposentadoria	divide responsabilidades		Sim
2017-10-01	60E. D.N.	Feminino		Ensino Superior		com filho(s)								Sim
2017-10-05	66E P R	Feminino				com filho(s)	Outros	Sim	Entre 53 a 60 anos		aposentadoria			Sim
2017-10-09	60C.M.F	Feminino	Divorciado(a)	Ensino Superior		com parentes outros	Professor(a)							Sim
2017-10-13	69CSG	Feminino	Divorciado(a)	Ensino Superior		sozinho	Professor(a)	Sim	Acima de 60 anos		aposentadoria			Sim
2017-10-14	64ESM	Feminino				sozinho	Do lar							Sim
2017-10-14	67RMND	Feminino	Divorciado(a)	Ensino Superior		sozinho	Outros	Sim	Entre 40 e 52 anos		aposentadoria			Sim
2017-10-16	74CMPA	Feminino		Ensino Superior		sozinho	Professor(a)	Sim	Entre 40 e 52 anos		aposentadoria e outros			Sim
2017-10-17	62MHBCK	Feminino	Casado(a)	Ensino Superior			Professor(a)	Sim	Entre 53 a 60 anos		aposentadoria			Sim
2017-10-20	68JNS	Masculino	Casado(a)	Ensino Superior			Financeiro/ Administrativo	Sim	Entre 40 e 52 anos		aposentadoria			Sim
2017-10-21	60I P L	Feminino	Solteiro(a)	Ensino Superior		com filho(s)					outros			Sim
2017-10-23	71Ceps	Feminino	Solteiro(a)	Ensino Superior		com parentes outros	Professor(a)	Sim	Entre 40 e 52 anos		aposentadoria	divide responsabilidades		Sim
2017-11-14	76GGL	Feminino	Solteiro(a)	Ensino Superior	outros	com netos	Outros		Acima de 60 anos	outros	aposentadoria	divide responsabilidades		Sim
2017-11-15	64VLLG	Feminino	Divorciado(a)	Ensino Superior		com filho(s)	Outros						sim	Sim
2017-11-20	61NTA	Feminino	Divorciado(a)		alugada	sozinho	Outros	Sim	Entre 53 a 60 anos		aposentadoria			Sim
2017-12-01	69MADSK	Feminino	Casado(a)	Ensino Superior		com companheiro(a) e filhos	Professor(a)	Sim	Entre 40 e 52 anos		aposentadoria			Sim
2018-02-08	69Ro	Feminino	Divorciado(a)		alugada	sozinho	Do lar							Sim
2018-02-13	69WHBM	Feminino			cedida	sozinho	Outros	Sim	Entre 53 a 60 anos	idade	aposentadoria			Sim
2018-02-13	60L m a	Feminino	Casado(a)	Ensino Superior	alugada		Outros	Sim	Entre 53 a 60 anos		aposentadoria	divide responsabilidades		Sim
2018-02-13	74A S H	Feminino				sozinho	Do lar	Sim	Acima de 60 anos		aposentadoria			Sim
2018-02-15	67A M O M	Feminino	Casado(a)				Professor(a)	Sim	Entre 53 a 60 anos		aposentadoria			Sim
2018-02-16	63TAB	Feminino	Solteiro(a)	Ensino Superior		com parentes outros	Professor(a)	Sim	Entre 40 e 52 anos		aposentadoria	divide responsabilidades		Sim
2018-02-26	71MCSN	Feminino	Solteiro(a)	Ensino Superior		com parentes outros	Outros	Sim	Entre 40 e 52 anos	outros	aposentadoria	divide responsabilidades		Sim
2018-02-26	68APF	Feminino	Divorciado(a)	Ensino Superior		com parentes outros	Outros	Sim	Entre 53 a 60 anos		aposentadoria			Sim
2018-03-01	61Gjgsm	Feminino	Casado(a)	Ensino Superior		sozinho	Do lar							Sim
2018-03-04	60mi	Feminino	Casado(a)				Do lar	Sim	Entre 53 a 60 anos	idade	aposentadoria e outros	divide responsabilidades		Sim
2018-03-11	60A	Feminino		Ensino Superior		com filho(s)	Professor(a)						sim	Sim
2018-03-11	64M C N M	Feminino	Casado(a)	Ensino Superior		com companheiro(a) e filhos	Professor(a)	Sim	Entre 53 a 60 anos		aposentadoria			Sim
2018-03-12	66Eac	Feminino	Casado(a)	Ensino Superior		com companheiro(a) e filhos	Do lar	Sim	Acima de 60 anos	idade	aposentadoria	divide responsabilidades		Sim
2018-03-13	62Gjgsm	Feminino	Casado(a)	Ensino Superior		sozinho	Do lar							Sim
2018-03-13	79Wa	Feminino		Ensino Superior			Outros	Sim	Acima de 60 anos		aposentadoria	divide responsabilidades		Sim
2018-03-13	70MM	Feminino	Casado(a)				Outros	Sim	Entre 53 a 60 anos		aposentadoria	divide responsabilidades		Sim
2018-03-14	65R	Feminino	Casado(a)	Ensino Superior			Professor(a)	Sim	Entre 53 a 60 anos		aposentadoria	divide responsabilidades		Sim
2018-03-16	65MTS	Feminino	Divorciado(a)			sozinho	Outros	Sim	Acima de 60 anos	idade	aposentadoria			Sim
2018-03-16	69NM	Feminino				com filho(s)	Professor(a)	Sim	Entre 53 a 60 anos		aposentadoria	divide responsabilidades		Sim
2018-03-17	64RJM	Masculino	Casado(a)	Ensino Superior			Professor(a)	Sim	Entre 53 a 60 anos			divide responsabilidades		Sim

2018-03-17	65	JWT	Masculino	Divorciado(a)	Ensino Superior		sozinho	Outros	Sim	Entre 53 a 60 anos		aposentadoria			Sim		
2018-03-18	77	V.A.	Feminino		Ensino Superior		sozinho	Outros	Sim	Entre 40 e 52 anos					Sim		
2018-03-19	71	JMOV	Feminino	Solteiro(a)	Ensino Superior		sozinho	Advogado(a)	Sim	Acima de 60 anos		aposentadoria			Sim		
2018-03-23	63	Mrg	Feminino	Casado(a)	Ensino Superior			Outros					divide responsabilidades		Sim		
2018-03-25	69	JOA	Masculino	Casado(a)	Ensino Superior			Professor(a)	Sim	Entre 53 a 60 anos		aposentadoria	divide responsabilidades		Sim		
2018-03-25	62	IEN	Feminino	Solteiro(a)	Ensino Superior		sozinho		Sim	Acima de 60 anos					Sim		
2017-09-25	68	M.A.M.J	Feminino				com filho(s)	Professor(a)	Sim	Entre 40 e 52 anos		aposentadoria	divide responsabilidades		Sim		
2017-09-28	73	IBAP	Feminino	Casado(a)				Outros	Sim	Entre 53 a 60 anos		aposentadoria	divide responsabilidades		Sim		
2017-10-01	69	MEMD	Feminino	Divorciado(a)	Ensino Superior		sozinho	Professor(a)	Sim	Entre 40 e 52 anos		aposentadoria			Sim		
2017-10-01	64	LBCSC	Feminino	Casado(a)	Ensino Superior				Sim	Entre 40 e 52 anos		aposentadoria	divide responsabilidades		Sim		
2017-05-07	64	L.B.C.S.C	Feminino	Casado(a)	Ensino Superior			Outros	Sim	Entre 40 e 52 anos		aposentadoria			Sim		
2017-10-08	65	ISR	Feminino	Divorciado(a)	Ensino Superior		sozinho	Financeiro/ Administrativo	Sim	Entre 53 a 60 anos		aposentadoria e outros			Sim		2,4,6,7
2017-12-09	65	ISR	Feminino	Divorciado(a)	Ensino Superior		sozinho	Financeiro/ Administrativo	Sim	Entre 53 a 60 anos	idade	aposentadoria e outros			Sim		2,4,6,7
2018-02-09	60	Ivani	Feminino	Casado(a)			com companheiros(a), filho(s) e neto(s)	Do lar				aposentadoria			Sim	7- Dor	
2018-02-13	65	V R S	Feminino	Solteiro(a)			sozinho	Outros	Sim	Acima de 60 anos		aposentadoria			Sim		2,4,6,9
2017-10-22	72	L.B.M	Feminino	Solteiro(a)	Ensino Superior		sozinho	Professor(a)	Sim	Acima de 60 anos		aposentadoria			Sim		2,4,6,8
2017-10-05	64	F.M.D.	Feminino	Casado(a)			com companheiro(a) e filhos	Do lar	Sim	Entre 53 a 60 anos	idade	aposentadoria	divide responsabilidades		Sim		4,7
2017-10-17	61	MLXA	Feminino	Casado(a)	Ensino Superior			Professor(a)	Sim	Entre 53 a 60 anos		aposentadoria	divide responsabilidades		Sim		2,4,7
2018-02-21	63	M	Feminino	Casado(a)				Do lar	Sim	Entre 53 a 60 anos	idade	aposentadoria	divide responsabilidades	sim	Sim		2,4,7
2018-03-12	69	VESV	Feminino	Casado(a)					Sim	Entre 53 a 60 anos	idade				Sim		2,4,7
2018-03-03	65	NMG	Feminino	Casado(a)	Ensino Superior			Professor(a)	Sim	Entre 40 e 52 anos		aposentadoria	divide responsabilidades		Sim	7- Dor, 12- Outros	7,12
2017-04-30	72	MS	Feminino	Solteiro(a)			com parentes outros	Outros	Sim	Entre 53 a 60 anos	idade	aposentadoria			Sim	3- Diabetes, 7- Dor	
2017-05-08	68	E S A S	Feminino	Casado(a)			com companheiro(a) e filhos	Do lar	Sim	Acima de 60 anos	idade		divide responsabilidades		Sim		
2017-10-23	62	T .O	Feminino	Casado(a)	Ensino Superior		com companheiro(a) e filhos					outros	divide responsabilidades		Sim	7- Dor	
2018-02-18	67	Jcb	Feminino		Ensino Superior	alugada	com filho(s)	Outros							Sim	7- Dor	
2018-03-02	61	MC	Feminino	Casado(a)	Ensino Superior			Professor(a)	Sim	Entre 53 a 60 anos		aposentadoria	divide responsabilidades		Sim	7- Dor	
2018-03-20	73	n p m	Feminino	Casado(a)	Ensino Superior			Professor(a)	Sim	Entre 40 e 52 anos		aposentadoria e outros	divide responsabilidades		Sim	7- Dor	
2018-03-11	63	MMCCV	Feminino	Casado(a)	Ensino Superior			Professor(a)	Sim	Entre 53 a 60 anos		aposentadoria	divide responsabilidades		Sim		2,3,4,6
2018-02-22	65	L A N D	Feminino	Casado(a)	Ensino Superior			Professor(a)	Sim	Entre 53 a 60 anos		aposentadoria	divide responsabilidades		Sim		3,6,12
2017-10-17	74	A C P	Feminino	Divorciado(a)	Ensino Superior		sozinho	Advogado(a)	Sim	Acima de 60 anos		outros			Sim		2,3,4,7
2017-10-23	70	AVV	Feminino		Ensino Superior		sozinho	Outros	Sim	Entre 53 a 60 anos	idade				Sim		2,4,5,7
2017-05-07	64	JM	Masculino	Casado(a)	Ensino Superior		com companheiro(a) e filhos	Outros	Sim	Acima de 60 anos		aposentadoria			Sim	3- Diabetes, 7- Dor	3,7
2017-05-03	70	RMES	Masculino	Casado(a)				Professor(a)	Sim	Entre 53 a 60 anos		aposentadoria			Sim	3- Diabetes, 5- Colesterol	3,5
2018-02-14	60	MTM	Feminino	Solteiro(a)	Ensino Superior		com parentes outros	Professor(a)	Sim	Entre 53 a 60 anos		aposentadoria	divide responsabilidades		Sim	3- Diabetes, 5- Colesterol	3,5
2017-09-27	80	S.T.S.B.	Feminino				sozinho	Professor(a)	Sim	Entre 40 e 52 anos					Sim		1,3,6,7
2017-10-14	61	DL	Feminino	Divorciado(a)		alugada	com filho(s)	Outros	Sim	Entre 53 a 60 anos		aposentadoria			Sim	3- Diabetes,12- Outros	3,12
2017-11-16	64	TCRP	Feminino		Ensino Superior		sozinho	Outros							Sim	3- Diabetes, 12- Outros	3,12
2017-04-30	63	e p f	Feminino	Casado(a)			com companheiro(a) e filhos	Do lar					divide responsabilidades	sim	Sim		
2017-05-18	65	NRS	Feminino		Ensino Superior		sozinho	Professor(a)	Sim	Entre 53 a 60 anos	idade				Sim	3- Diabetes	
2017-11-14	63	M. E. C. V.	Feminino	Casado(a)	Ensino Superior	alugada	sozinho	Professor(a)	Sim	Acima de 60 anos		aposentadoria			Sim	3- Diabetes	
2018-02-11	62	S	Feminino		Ensino Superior		com parentes outros	Advogado(a)				aposentadoria e outros		sim	Sim	3- Diabetes	

2018-03-03	64S	Feminino	Divorciado(a)	Ensino Superior		com parentes outros		Sim	Entre 53 a 60 anos	idade			sim	Sim	3- Diabetes	
2018-03-12	61JSC	Masculino	Casado(a)			com companheiro(a) e filhos	Outros	Sim	Entre 53 a 60 anos		aposentadoria	divide responsabilidades	sim	Sim	3- Diabetes	
2017-10-01	61VLML	Feminino	Casado(a)	Ensino Superior	alugada			Sim	Entre 53 a 60 anos		aposentadoria			Sim	3- Diabetes	
2017-05-17	73DS	Feminino	Casado(a)	Ensino Superior		com parentes outros	Professor(a)	Sim	Entre 53 a 60 anos		outros			Sim		2,4,12
2017-09-30	60v	Feminino	Casado(a)		alugada		Do lar				outros			Sim		2,4,12
2018-03-02	61Irani	Feminino				com filho(s)		Sim	Entre 53 a 60 anos	idade		divide responsabilidades		Sim		2,4,12
2017-09-29	69CD	Feminino	Casado(a)	Ensino Superior			Profissional liberal	Sim	Entre 53 a 60 anos		aposentadoria	divide responsabilidades		Sim		2,4
2017-10-12	62MIA	Feminino		Ensino Superior			Professor(a)	Sim	Entre 53 a 60 anos		aposentadoria	divide responsabilidades		Sim		2,4
2017-11-13	60SAA	Feminino				sozinho	Professor(a)	Sim	Entre 40 e 52 anos		aposentadoria			Sim		2,4
2017-11-20	60LRSF	Feminino				com filho(s)	Outros				aposentadoria			Sim		2,4
2017-12-01	60Eacf	Feminino	Divorciado(a)	Ensino Superior		sozinho								Sim		2,4
2018-02-12	61NFV	Feminino	Divorciado(a)		cedida	sozinho	Outros	Sim	Entre 53 a 60 anos		aposentadoria			Sim		2,4
2018-02-13	62MHL	Feminino	Casado(a)			sozinho	Outros	Sim	Entre 53 a 60 anos	idade	aposentadoria			Sim		2,4
2018-02-14	70T M	Feminino	Solteiro(a)	Ensino Superior		sozinho	Professor(a)	Sim	Entre 40 e 52 anos		aposentadoria			Sim		2,4
2018-02-15	61MM	Feminino	Solteiro(a)	Ensino Superior		sozinho		Sim	Entre 53 a 60 anos		aposentadoria e outros			Sim		2,4
2018-02-28	68m l g	Feminino		Ensino Superior		sozinho	Professor(a)	Sim	Acima de 60 anos		aposentadoria			Sim		2,4
2018-03-05	61MDR	Feminino	Divorciado(a)		alugada	com filho(s)	Do lar							Sim		2,4
2017-09-29	69Mrb	Feminino		Ensino Superior		sozinho	Do lar	Sim	Entre 40 e 52 anos		aposentadoria			Sim		2,4
2018-03-15	67I. M. O	Feminino				sozinho	Do lar							Sim		1,5,10
2018-03-13	61FFDM	Feminino	Divorciado(a)	Ensino Superior		sozinho	Professor(a)							Sim		5,7,8,[illegible]
2018-03-06	73CVR	Feminino	Divorciado(a)			sozinho	Outros	Sim	Acima de 60 anos	idade	aposentadoria			Sim	5- Colesterol, 8- Reumatismo	5,8
2017-10-09	65Edna	Feminino				sozinho	Financeiro/ Administrativo							Sim		2,4,5,[illegible]
2018-03-04	61N M	Feminino	Divorciado(a)	Ensino Superior	cedida	sozinho	Outros							Sim		2,4,5,[illegible]
2017-09-28	67D M M	Feminino	Casado(a)	Ensino Superior				Sim	Entre 40 e 52 anos		aposentadoria e outros			Sim		5,6
2017-09-26	63CCM	Feminino	Casado(a)	Ensino Superior			Outros	Sim	Entre 53 a 60 anos	outros	outros			Sim		2,4,5,[illegible]
2018-03-22	65mlv	Feminino	Divorciado(a)	Ensino Superior		com filho(s)	Do lar							Sim		2,4,5,[illegible]
2017-10-12	62GPS	Feminino	Casado(a)	Ensino Superior		com companheiros(a), filho(s) e neto(s)	Outros					divide responsabilidades		Sim		2,4,5
2017-10-12	60A M D	Feminino	Divorciado(a)	Ensino Superior		com filho(s)	Professor(a)	Sim	Entre 53 a 60 anos		aposentadoria			Sim		2,4,5
2017-10-14	70I O C	Feminino	Casado(a)				Professor(a)	Sim	Entre 53 a 60 anos		aposentadoria			Sim		2,4,5
2017-10-18	62A m d	Feminino	Divorciado(a)	Ensino Superior		com filho(s)	Professor(a)	Sim	Entre 53 a 60 anos		aposentadoria			Sim		2,4,5
2018-02-23	69DGC	Masculino				sozinho	Outros	Sim	Acima de 60 anos		aposentadoria		sim	Sim		2,4,5
2017-10-01	60S.A.S.	Feminino				sozinho	Professor(a)	Sim	Entre 40 e 52 anos		aposentadoria e outros			Sim		2,4,5
2017-10-09	63MM	Feminino	Casado(a)	Ensino Superior			Outros	Sim	Entre 40 e 52 anos		aposentadoria	divide responsabilidades		Sim	5- Colesterol, 12- Outros	5,12
2017-10-14	60E T S	Feminino	Casado(a)				Professor(a)	Sim	Entre 40 e 52 anos		aposentadoria			Sim	5- Colesterol, 12- Outros	5,12
2017-10-14	74ARF	Feminino	Solteiro(a)			com parentes outros	Professor(a)	Sim	Entre 53 a 60 anos		aposentadoria			Sim	5- Colesterol, 12- Outros	5,12
2017-11-22	64ME	Feminino	Casado(a)	Ensino Superior			Do lar	Sim	Acima de 60 anos	idade	aposentadoria			Sim	5- Colesterol, 12- Outros	5,12
2018-03-07	63M.I.F	Feminino	Divorciado(a)		alugada	com parentes outros	Do lar					divide responsabilidades		Sim	5- Colesterol, 12- Outros	5,12
2018-03-07	63L. A. H.	Feminino	Casado(a)				Professor(a)	Sim	Entre 40 e 52 anos		aposentadoria	divide responsabilidades		Sim	5- Colesterol, 12- Outros	5,12
2017-04-29	73IBAP	Feminino	Casado(a)				Outros	Sim	Entre 53 a 60 anos		aposentadoria e outros	divide responsabilidades		Sim		
2017-05-21	67Mjpb	Feminino	Divorciado(a)			com companheiro(a) e filhos		Sim	Entre 53 a 60 anos	idade	aposentadoria e outros			Sim	5- Colesterol	

2017-10-16	M C B M 60G	Feminino	Casado(a)	Ensino Superior			Engenheiro(a)	Sim	Entre 40 e 52 anos	aposentadoria especial		divide responsabilidades	sim	Sim	5- Colesterol
2017-10-24	78ISV	Feminino		Ensino Superior	alugada	sozinho		Sim	Entre 40 e 52 anos		aposentadoria e outros			Sim	5- Colesterol
2017-11-15	60GSL	Masculino	Divorciado(a)	Ensino Superior	alugada	com filho(s)	Professor(a)	Sim	Entre 53 a 60 anos					Sim	5- Colesterol
2018-03-21	61Hdcl	Feminino	Casado(a)			com companheiro(a) e filhos		Sim	Entre 53 a 60 anos		aposentadoria	divide responsabilidades		Sim	5- Colesterol
2017-09-25	63Rcf	Feminino	Casado(a)	Ensino Superior				Sim	Entre 53 a 60 anos		aposentadoria e outros	divide responsabilidades		Sim	5- Colesterol
2017-10-01	70Ecs	Feminino				com filho(s)		Sim	Entre 53 a 60 anos	idade	aposentadoria	divide responsabilidades		Sim	5- Colesterol
2017-05-05	61ABS	Feminino	Casado(a)	Ensino Superior			Do lar					divide responsabilidades		Sim	7- Dor, , 12- Outros
2017-05-19	63RM	Feminino	Divorciado(a)	Ensino Superior		sozinho	Professor(a)	Sim	Entre 53 a 60 anos		aposentadoria			Sim	12- Outros
2017-09-30	61RB	Feminino	Casado(a)	Ensino Superior			Outros	Sim	Entre 40 e 52 anos		aposentadoria	divide responsabilidades		Sim	12- Outros
2017-10-09	73m.m.a.g.f.	Feminino	Casado(a)				Outros	Sim	Acima de 60 anos		aposentadoria	divide responsabilidades		Sim	12- Outros
2017-10-10	60J	Feminino	Casado(a)			com companheiro(a) e filhos	Outros	Sim	Entre 53 a 60 anos		aposentadoria	divide responsabilidades		Sim	12- Outros
2017-10-13	70R. M.	Feminino	Casado(a)	Ensino Superior			Professor(a)	Sim	Entre 53 a 60 anos	idade	aposentadoria	divide responsabilidades	sim	Sim	12- Outros
2017-10-14	73MMLSMBEH	Feminino	Casado(a)	Ensino Superior			Professor(a)	Sim	Entre 53 a 60 anos		aposentadoria	divide responsabilidades		Sim	12- Outros
2017-10-15	64LFM	Feminino		Ensino Superior		com parentes outros	Do lar				outros			Sim	12- Outros
2017-10-18	65S M S P	Feminino	Casado(a)	Ensino Superior		com parentes outros		Sim	Entre 53 a 60 anos		aposentadoria	divide responsabilidades		Sim	12- Outros
2017-10-22	60MJFS	Feminino	Solteiro(a)	Ensino Superior		sozinho								Sim	12- Outros
2017-10-23	75Bf	Feminino		Ensino Superior		sozinho		Sim	Acima de 60 anos		outros	divide responsabilidades		Sim	12- Outros
2017-11-12	64NSV	Feminino	Divorciado(a)			com parentes outros	Outros	Sim	Entre 53 a 60 anos	idade	aposentadoria e outros	divide responsabilidades		Sim	12- Outros
2017-11-12	62CSA	Feminino	Divorciado(a)			com filho(s)	Outros					divide responsabilidades		Sim	12- Outros
2017-11-16	61Mlmf	Feminino		Ensino Superior		com filho(s)	Outros	Sim	Entre 40 e 52 anos		aposentadoria	divide responsabilidades	sim	Sim	12- Outros
2017-11-18	73Mgsa	Feminino	Casado(a)	Ensino Superior			Outros	Sim	Entre 53 a 60 anos		aposentadoria	divide responsabilidades		Sim	12- Outros
2017-11-21	62M.T	Feminino	Divorciado(a)	Ensino Superior		sozinho	Professor(a)	Sim	Acima de 60 anos		aposentadoria			Sim	12- Outros
2017-11-28	74MEMT	Feminino	Casado(a)			com companheiro(a) e filhos	Do lar	Sim	Acima de 60 anos	aposentadoria especial	aposentadoria	divide responsabilidades		Sim	12- Outros
2017-11-29	74MEMT	Feminino	Casado(a)			com companheiro(a) e filhos	Do lar	Sim	Acima de 60 anos	aposentadoria especial	aposentadoria	divide responsabilidades		Sim	12- Outros
2017-12-01	60AOS	Feminino	Casado(a)	Ensino Superior	alugada	com companheiro(a) e filhos						divide responsabilidades		Sim	12- Outros
2018-02-09	66V	Feminino	Casado(a)	Ensino Superior			Outros	Sim	Acima de 60 anos		aposentadoria e outros	divide responsabilidades		Sim	12- Outros
2018-02-11	65ACBS	Feminino	Casado(a)	Ensino Superior			Professor(a)	Sim	Acima de 60 anos		aposentadoria			Sim	12- Outros
2018-02-12	62IMF	Feminino	Divorciado(a)			sozinho	Do lar	Sim	Entre 53 a 60 anos	idade	aposentadoria			Sim	12- Outros
2018-02-13	61MD	Feminino	Solteiro(a)	Ensino Superior	cedida	com filho(s)	Professor(a)	Sim	Entre 53 a 60 anos		aposentadoria			Sim	12- Outros
2018-02-13	75Fz	Feminino		Ensino Superior		sozinho	Professor(a)	Sim	Entre 40 e 52 anos					Sim	12- Outros
2018-02-13	62MCZ	Feminino		Ensino Superior	alugada	com filho(s)	Outros	Sim	Entre 53 a 60 anos				sim	Sim	12- Outros
2018-02-16	66DTC.	Feminino		Ensino Superior		sozinho	Outros	Sim	Acima de 60 anos	idade	aposentadoria			Sim	12- Outros
2018-02-21	64AN	Feminino	Solteiro(a)	Ensino Superior		sozinho	Outros	Sim	Entre 53 a 60 anos		outros	divide responsabilidades		Sim	12- Outros
2018-02-21	60E-E	Feminino	Divorciado(a)	Ensino Superior		sozinho	Outros							Sim	12- Outros
2018-02-25	60MRHZ	Feminino	Casado(a)				Outros	Sim	Entre 53 a 60 anos		aposentadoria	divide responsabilidades	sim	Sim	12- Outros
2018-02-28	67GMMS	Feminino	Solteiro(a)	Ensino Superior	alugada	sozinho	Outros	Sim	Entre 53 a 60 anos		aposentadoria			Sim	12- Outros
2018-03-04	60B.O	Feminino	Divorciado(a)		alugada		Outros	Sim	Entre 53 a 60 anos		aposentadoria	divide responsabilidades		Sim	12- Outros
2018-03-04	69ES	Feminino				sozinho		Sim	Acima de 60 anos	idade				Sim	12- Outros
2018-03-04	64TCRP	Feminino		Ensino Superior		sozinho	Profissional liberal							Sim	12- Outros

2018-03-06	61	Jaeh	Masculino	Casado(a)	Ensino Superior			Professor(a)		Entre 53 a 60 anos		aposentadoria e outros			Sim	12- Outros
2018-03-07	62	M	Feminino	Casado(a)	Ensino Superior			Professor(a)	Sim	Entre 53 a 60 anos		aposentadoria	divide responsabilidades		Sim	12- Outros
2018-03-11	74	ITC	Feminino	Casado(a)	Ensino Superior			Outros	Sim	Entre 40 e 52 anos		aposentadoria	divide responsabilidades		Sim	12- Outros
2018-03-12	75	MES	Feminino	Casado(a)	Ensino Superior			Professor(a)	Sim	Entre 53 a 60 anos		aposentadoria	divide responsabilidades		Sim	12- Outros
2018-03-13	63	RL	Feminino	Divorciado(a)		alugada	com filho(s)	Outros	Sim	Entre 53 a 60 anos	outros	aposentadoria			Sim	12- Outros
2018-03-14	64	Mim	Feminino		Ensino Superior		sozinho	Professor(a)	Sim	Entre 40 e 52 anos		aposentadoria			Sim	12- Outros
2018-03-15	62	M	Feminino	Divorciado(a)	Ensino Superior		com filho(s)	Outros	Sim	Entre 53 a 60 anos	outros				Sim	12- Outros
2018-03-18	60	VS	Feminino	Casado(a)				Professor(a)	Sim	Entre 53 a 60 anos	idade	aposentadoria	divide responsabilidades		Sim	12- Outros
2018-03-19	65	MFPB	Feminino		Ensino Superior		sozinho								Sim	12- Outros
2017-04-29	70	T G	Feminino				sozinho	Professor(a)	Sim	Acima de 60 anos		aposentadoria				
2017-05-05	64	IHR	Feminino	Casado(a)	Ensino Superior		com companheiro(a) e filhos	Professor(a)	Sim	Entre 40 e 52 anos			divide responsabilidades	sim		
2017-05-07	64	GAT	Feminino		Ensino Superior		sozinho	Outros	Sim	Entre 53 a 60 anos		aposentadoria				
2017-05-15	74	hmgr	Feminino	Casado(a)	Ensino Superior			Professor(a)	Sim	Entre 40 e 52 anos		aposentadoria	divide responsabilidades			
2017-05-17	65	VLA	Feminino	Casado(a)	Ensino Superior	outros	com companheiro(a) e filhos									
2017-05-18	61	sim	Feminino					Outros	Sim	Entre 40 e 52 anos		aposentadoria				
2017-10-06	77	THG	Feminino	Casado(a)	Ensino Superior			Professor(a)	Sim	Entre 53 a 60 anos		aposentadoria	divide responsabilidades			
2017-10-07	63	SMC	Feminino			alugada	com filho(s)	Do lar				outros				
2017-10-12	69	MSSS	Feminino	Casado(a)	Ensino Superior			Professor(a)	Sim	Entre 40 e 52 anos		aposentadoria				
2017-10-12	62	Emjr	Feminino	Casado(a)	Ensino Superior			Professor(a)					divide responsabilidades			
2017-10-14	60	MGAM	Feminino	Casado(a)			com parentes outros					outros	divide responsabilidades			
2017-10-15	61	ETM	Feminino	Solteiro(a)	Ensino Superior	alugada	sozinho	Outros	Sim	Entre 53 a 60 anos		aposentadoria		sim		
2017-10-20	69	V R H V	Feminino	Casado(a)	Ensino Superior			Professor(a)	Sim	Entre 40 e 52 anos		aposentadoria e outros				
2017-10-20	73	D O	Feminino	Divorciado(a)	Ensino Superior	alugada	com filho(s)	Financeiro/ Administrativo					divide responsabilidades			
2017-10-22	61	E N	Feminino	Casado(a)			com companheiro(a) e filhos	Outros					divide responsabilidades			
2017-10-23	64	V LM	Feminino	Casado(a)			com companheiro(a) e filhos	Professor(a)	Sim	Entre 40 e 52 anos		aposentadoria	divide responsabilidades			
2017-10-23	65	JDN	Masculino		Ensino Superior		sozinho	Financeiro/ Administrativo	Sim	Entre 53 a 60 anos		aposentadoria				
2017-10-23	60	AESQ	Feminino	Casado(a)	Ensino Superior			Outros	Sim	Entre 53 a 60 anos	idade	aposentadoria	divide responsabilidades			
2017-10-24	66	PPB	Masculino	Casado(a)	Ensino Superior	alugada		Outros	Sim	Entre 40 e 52 anos		aposentadoria				
2017-11-12	67	I c	Feminino				com filho(s)	Profissional liberal	Sim	Entre 53 a 60 anos	idade	aposentadoria				
2017-11-12	65	Ilza	Feminino		Ensino Superior		com parentes outros	Outros	Sim	Acima de 60 anos		aposentadoria e outros	divide responsabilidades			
2017-11-13	60	GR	Feminino	Divorciado(a)			com parentes outros	Outros	Sim	Entre 53 a 60 anos		outros	divide responsabilidades			
2017-11-13	60	SM	Feminino	Solteiro(a)	Ensino Superior	alugada	sozinho	Engenheiro(a)	Sim	Entre 53 a 60 anos		aposentadoria				
2017-11-13	71	METB	Feminino	Casado(a)	Ensino Superior			Professor(a)	Sim	Entre 40 e 52 anos		aposentadoria	divide responsabilidades			
2017-11-15	68	MQAA	Feminino	Casado(a)			com companheiro(a) e filhos	Professor(a)	Sim	Entre 40 e 52 anos		aposentadoria	divide responsabilidades			
2017-11-20	65	SPS	Feminino	Divorciado(a)	Ensino Superior	alugada	sozinho	Profissional liberal	Sim	Acima de 60 anos		aposentadoria		sim		
2017-11-21	65	AL	Feminino	Divorciado(a)	Ensino Superior		sozinho	Do lar				outros				
2017-11-22	69	TMGS	Feminino	Casado(a)				Professor(a)	Sim	Entre 53 a 60 anos		aposentadoria	divide responsabilidades			
2017-11-26	61	WJF	Feminino	Casado(a)			com companheiro(a) e filhos	Outros				outros				
2017-12-03	67	J.M.M.	Feminino	Solteiro(a)			sozinho	Outros	Sim	Entre 40 e 52 anos		aposentadoria		sim		
2017-12-03	61	c	Feminino	Casado(a)			sozinho	Professor(a)	Sim	Entre 53 a 60 anos		aposentadoria e outros				

2018-02-09	69	M.E	Feminino	Casado(a)	Ensino Superior		com companheiro(a) e filhos	Do lar	Sim	Acima de 60 anos	idade	outros			
2018-02-10	79	MPB	Feminino	Solteiro(a)	Ensino Superior	alugada	sozinho	Professor(a)	Sim	Acima de 60 anos		aposentadoria			
2018-02-10	69	GMO	Feminino	Divorciado(a)	Ensino Superior	alugada	sozinho	Professor(a)	Sim	Entre 53 a 60 anos		aposentadoria	divide responsabilidades	sim	
2018-02-14	62	A M C	Feminino	Divorciado(a)	Ensino Superior	alugada	sozinho	Professor(a)							
2018-02-15	74	C C G	Feminino	Divorciado(a)	Ensino Superior		sozinho	Professor(a)	Sim	Entre 53 a 60 anos		aposentadoria			
2018-02-15	60	MC	Feminino	Solteiro(a)	Ensino Superior	alugada	com parentes outros	Professor(a)	Sim	Entre 53 a 60 anos	idade	aposentadoria e outros	divide responsabilidades	sim	
2018-02-16	62	Sl	Feminino		Ensino Superior		sozinho	Outros	Sim	Entre 53 a 60 anos		aposentadoria		sim	
2018-02-20	69	RMP	Masculino		Ensino Superior		sozinho	Outros	Sim	Entre 53 a 60 anos		aposentadoria			
2018-02-23	71	AKF	Masculino	Casado(a)	Ensino Superior			Outros	Sim	Entre 40 e 52 anos	aposentadoria especial	aposentadoria			
2018-02-24	70	MILP	Feminino	Casado(a)	Ensino Superior			Outros	Sim	Entre 53 a 60 anos		aposentadoria			
2018-02-25	62	NA	Feminino	Casado(a)	Ensino Superior			Professor(a)	Sim	Entre 40 e 52 anos		aposentadoria			
2018-03-04	60	NC	Feminino	Divorciado(a)	Ensino Superior		sozinho								
2018-03-04	67	V	Feminino	Casado(a)	Ensino Superior			Professor(a)	Sim	Acima de 60 anos	idade	aposentadoria	divide responsabilidades		
2018-03-05	65	A L L	Feminino	Divorciado(a)			sozinho	Do lar							
2018-03-06	69	ESC	Feminino	Casado(a)	Ensino Superior			Professor(a)				outros	divide responsabilidades		
2018-03-06	63	RVL	Feminino	Solteiro(a)	Ensino Superior		com filho(s)	Professor(a)							
2018-03-08	60	NC	Feminino		Ensino Superior		sozinho	Profissional liberal							
2018-03-08	62	Cm	Masculino	Casado(a)		outros		Profissional liberal							
2018-03-10	61	IG	Feminino	Divorciado(a)	Ensino Superior		sozinho					outros			
2018-03-11	61	M A	Feminino	Casado(a)			com parentes outros	Professor(a)					divide responsabilidades		
2018-03-12	64	AASR	Feminino			alugada	sozinho	Do lar					divide responsabilidades	sim	
2018-03-12	61	GAM	Feminino	Solteiro(a)	Ensino Superior		com parentes outros	Outros	Sim	Acima de 60 anos		aposentadoria	divide responsabilidades		
2018-03-15	74	RMGS	Feminino			cedida	com filho(s)	Profissional liberal	Sim	Acima de 60 anos	idade	aposentadoria	divide responsabilidades		
2018-03-17	61	R A S.	Feminino		Ensino Superior		com parentes outros	Professor(a)	Sim	Acima de 60 anos	idade	aposentadoria		sim	Sim
2018-03-10	60	R.B.	Feminino	Casado(a)			com companheiro(a) e filhos	Outros					divide responsabilidades		Sim
2018-03-17	63	RP	Feminino	Solteiro(a)	Ensino Superior		com parentes outros	Outros	Sim	Entre 53 a 60 anos			divide responsabilidades		
2018-03-17	61	E M P	Feminino	Casado(a)					Sim	Entre 53 a 60 anos	idade	aposentadoria			
2018-03-18	60	I A F	Feminino	Casado(a)	Ensino Superior			Outros	Sim	Entre 53 a 60 anos		aposentadoria	divide responsabilidades		
2018-03-19	66	M J P C	Masculino	Casado(a)	Ensino Superior		com companheiro(a) e filhos	Professor(a)	Sim	Acima de 60 anos		aposentadoria	divide responsabilidades		
2018-03-21	65	MC	Feminino	Solteiro(a)	Ensino Superior		com filho(s)	Outros	Sim	Acima de 60 anos	idade	aposentadoria			
2018-03-22	68	MC	Feminino		Ensino Superior		com filho(s)	Professor(a)				outros			
2018-03-22	63	MABL	Feminino	Solteiro(a)	Ensino Superior		com filho(s)	Professor(a)				outros			
2018-03-23	82	N. F. K.	Feminino				sozinho	Do lar							
2018-03-23	62	R	Feminino	Casado(a)			com companheiro(a) e filhos	Do lar		Acima de 60 anos	idade			sim	
2018-03-24	60	Lmpm.	Feminino	Casado(a)	Ensino Superior		com companheiros(a), filho(s) e neto(s)								
2018-03-24	76	B T	Feminino		Ensino Superior		sozinho	Do lar	Sim	Acima de 60 anos	outros	aposentadoria			
2017-09-28	60	MFN	Feminino	Divorciado(a)	Ensino Superior		com filho(s)	Professor(a)	Sim	Entre 53 a 60 anos		aposentadoria e outros		sim	
2017-10-02	61	SMFC	Feminino	Casado(a)	Ensino Superior			Financeiro/ Administrativo	Sim	Entre 53 a 60 anos	idade	aposentadoria	divide responsabilidades	sim	

2017-10-08	63FCG	Feminino	Divorciado(a)	Ensino Superior		com filho(s)	Do lar	Sim	Acima de 60 anos	idade	aposentadoria	divide responsabilidades	
2017-10-08	66R.P.	Masculino	Casado(a)				Profissional liberal						
2017-05-04	62MFRO	Feminino	Casado(a)		alugada		Outros						
2017-09-25	60RGABM	Feminino				sozinho	Professor(a)	Sim	Entre 53 a 60 anos		aposentadoria e outros		Sim
